# Distal Abdominal Aortic Thrombus With Approximately Full Occlusion—A Case of Pre‐Endovascular Care in Resource‐Limited Settings

**DOI:** 10.1002/ccr3.70759

**Published:** 2025-08-03

**Authors:** Hilary Chipongo, Abizer Sarkar, Esmail Sangey, Kishan Chudasama, Amar Swali, Shaffin Rajan

**Affiliations:** ^1^ Critical Care Department Shree Hindu Mandal Hospital Dar es Salaam Tanzania; ^2^ Radiology Department Shree Hindu Mandal Hospital Dar es Salaam Tanzania; ^3^ Department of Internal Medicine Shree Hindu Mandal Hospital Dar es Salaam Tanzania

**Keywords:** distal abdominal aortic thrombus, intra‐arterial vasodilator, papaverine, thrombolytics

## Abstract

Up‐to‐date management of a distal abdominal aortic thrombus is challenging as no dedicated guideline for treating this condition exists. In our setting, it is one of the rare arterial vascular emergencies that can lead to devastating consequences and an increase in patient morbidity or mortality. For this publication, we have a case report of an elderly female of Asian descent who failed multiple attempts of conservative management with thrombolytics but benefited from an intra‐arterial papaverine infusion to salvage her ischemic lower limb.

**Summary**

- Aortic thrombus is a rare and life‐threatening condition.
- Few guidelines are proposed for its management, as few cases are reported in the literature.
- Herein we present a deadly occlusion of the distal abdominal aorta, which was managed conservatively as a pre‐endovascular care in a resource‐limited setting.

**Abbreviations**
APTT: activated partial thromboplastin time
CT‐SCAN: computed tomography scan
INR: international normalized ratio
MRI: magnetic resonance imaging

## 1 Introduction

Distal abdominal aortic thrombus is one of the rare entities in cardiovascular pathologies [1], and being spontaneous makes it unique. Only a few cases are encountered in clinical practice; thus, it is rare to diagnose before the patient suffers embolic complications. This makes the true incidence of this particular disease unknown [2] and therefore a high index of suspicion in patients without risk of coagulability states or any previous risk factors predisposing them to thrombus formation should be thought of, especially in patients presenting with signs of lower extremity ischemia which may mimic signs of venous insufficiency due to deep vein thrombosis [3]. The management guidelines for this condition are still lacking as there is no clear consensus on whether conservative pharmacological versus endovascular surgery is the best option [3]. These guidelines are referenced from developed countries, which possess the full availability of resources and equipment, as opposed to our medical setting. In this case, we present a 69‐year‐old female of Asian descent who was diagnosed with distal aortic occlusion of approximately more than ninety percent (90%) and was managed with intra‐arterial papaverine and streptokinase infusion before being referred for endovascular therapy to salvage the limb.

## 2 Case Presentation

A 69‐year‐old female patient of Asian descent presented to the emergency department with a complaint of sudden onset of left calf pain approximately 12 h before admission. Upon further inquiry, she also reported having experienced numbness in her left lower limb, which was progressively worsening in the past 4 days before admission. Her only reported chronic comorbidity was Type 2 diabetes mellitus, for which she was on regular oral medications. On physical examination at the emergency department, she had a positive Homan's sign, dorsiflexion of the left foot produced significant calf pain, and dorsalis pedis and posterior tibial pulses were faint. A 12‐lead ECG (electrocardiography) showed an incidental atrial fibrillation (chronic) because a previous ECG done months earlier was similar.

## 3 Investigations

**FIGURE 1:**
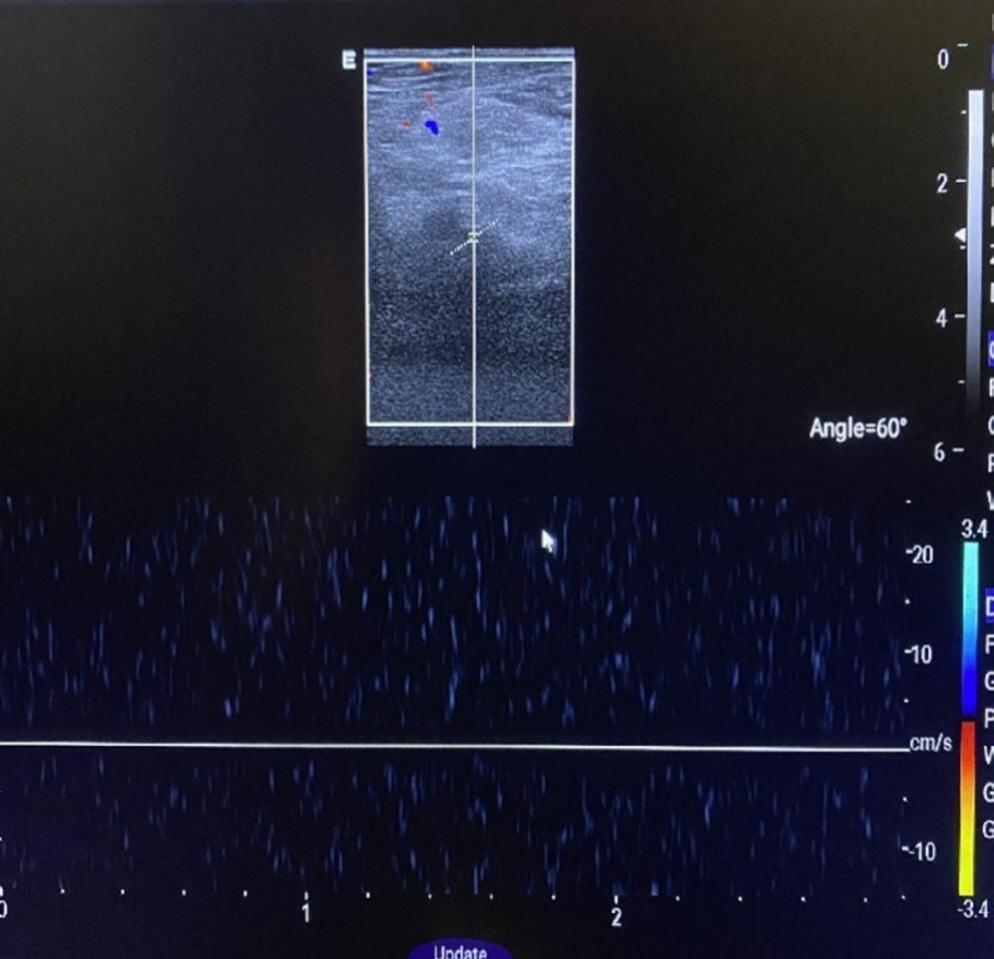
Doppler ultrasound of the left lower limb showed no color and spectral Doppler waveform of the popliteal artery suggestive of thrombus versus embolism with undetectable Doppler distal flow.

**FIGURE 2:**
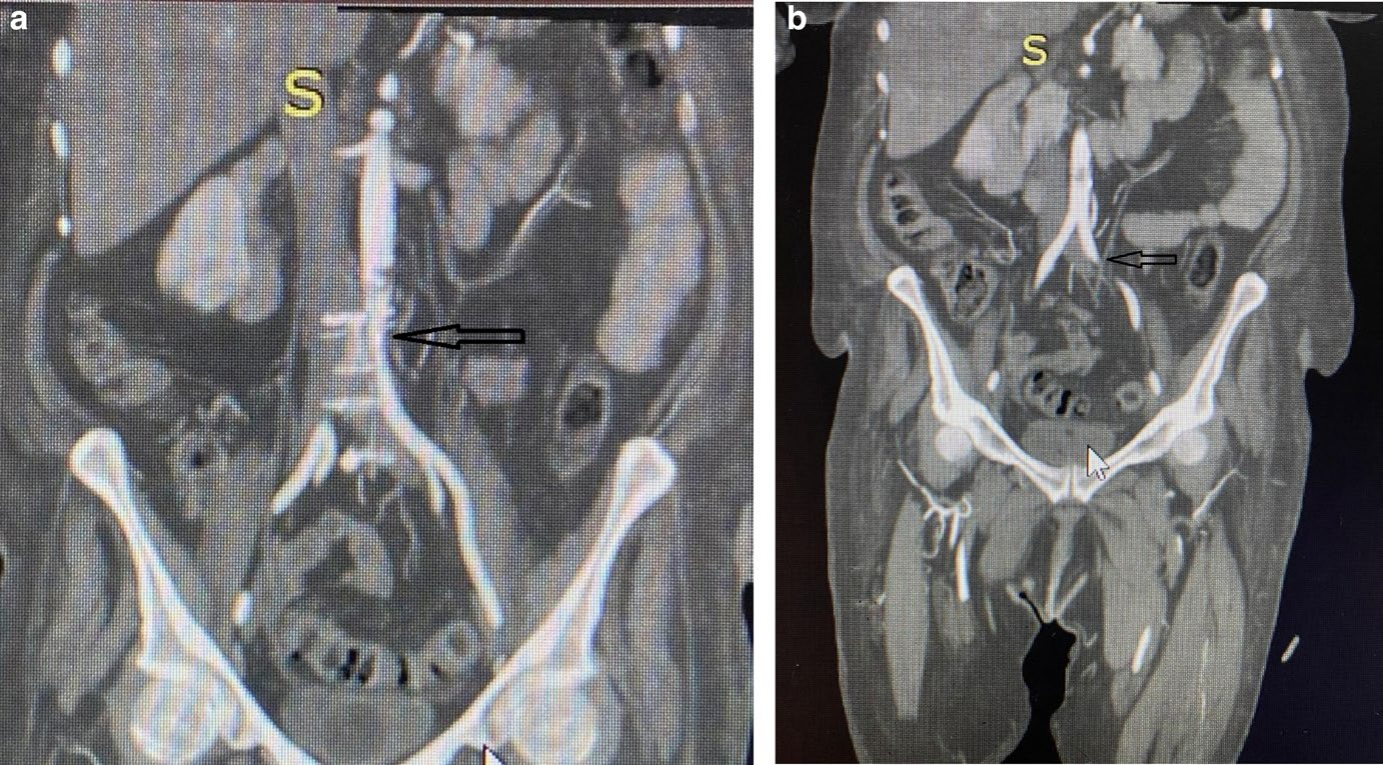
(a) A coronal view of contrast‐enhanced CT angiography showing an approximately full occlusion of the distal abdominal aorta black arrow. (b) A black arrow indicates left iliac artery filling defect.

Laboratory investigations revealed HbA1c of 5.56%, D‐dimer of 2.47, a negative rheumatoid factor, APTT 66.8, and INR 1.32. All other blood investigations were within the normal parameters. Doppler ultrasound of the left lower limb showed no vascular blood flow. A spectral Doppler waveform of the popliteal artery suggested thrombotic versus embolic occlusion with an undetectable Doppler distal flow (Figure 1). The patient was thrombosed with tenecteplase 30 mg and transferred to the ICU for monitoring. Second day post admission, the patient's left lower limb was found to be cyanotic at the medial toe and was cold and tender on palpation. No palpable foot pulse and pulse oximetry of the toes yielded no measurable plethysmograph, and no saturation could be recorded. The initial range of mobility of the toe at this level was significantly reduced; the patient could no longer flex her toes. An urgent CT angiography was done which showed a thrombus occluding approximately 90% of the distal abdominal aorta (Figure 2a). Figure 2b shows the absence of blood flow with a filling defect in the left iliac artery.

## 4 Results

The patient was given a loading dose of 250,000 IU of intravenous streptokinase followed by an infusion of 50,000 IU/h. Despite this, no clinical improvement was noted. Following a failure of this treatment, a decision was made to insert an arterial catheter in the left femoral artery, which was used to infuse medications directly into the obstruction. An intra‐arterial infusion of papaverine at 30 mg/h and streptokinase at 50,000 IU/h was initiated. Approximately 6 h after the initiation of this treatment, cyanosis of the left toe reduced significantly. The posterior tibial pulse could be palpated and pulse oximetry yielded a plethysmograph with saturations ranging from 50% to 69%. This treatment continued for 48 h and improved her left lower limb. The patient was thereafter referred to a higher facility for a vascular surgeon review and possible vascular stenting. Our country lacks the appropriate vascular stents required for such procedures. This was the justification for using intra‐arterial pharmacological therapy to salvage and maintain the arterial integrity of the limb until definitive care could be obtained.

## 5 Conclusion

Distal abdominal aortic thrombus is still a rare and often poorly diagnosed cardiovascular emergency that requires prompt management. Timely referral in resource‐limited settings is crucial to reduce morbidity and mortality in such patients. Treatment guidelines or protocols are still lacking for this particular condition, especially for patients who present in set‐ups with a lack of vascular surgeons and interventional devices such as stents. Taking note of the case we encountered, in surgical interventional resource‐limited settings like ours, timely diagnosis and aggressive pharmacological therapy remain the only options preceding a referral.

## 6 Discussion

These cases mostly present with manifestations of thromboembolism and can mostly be asymptomatic [4]. Common symptoms seen in the literature are extremity pain, skin discolouration, gangrene, and necrosis. These are the most common initial presentation symptoms accounting for more than 45% of cases [4]. Risk factors such as peripheral arterial diseases, autoimmune disorders, poorly controlled glycemic levels, and hypercoagulability states (Virchow's triad) have been reported to increase the risk of arterial thrombosis formation [5]. In our patient, the notable clinical presentation was calf pain, preceded by numbness of the left lower limb 4 days before admission.

Several modalities of diagnosis of distal abdominal aortic thrombus have been used in clinical practice, including echocardiography, computed tomography, MRI (magnetic resonance imaging), and angiography [4]. Up‐to‐date CT angiography is the investigation of choice and has been used in approximately 78% of cases to diagnose distal abdominal aortic thrombus [4, 6]. Prompt diagnosis is still a challenge, and clinical judgment is of paramount importance to even consider the possibility of an arterial thrombosis, as it is likely to be rare in distal regions without affecting axial parts of the body. Literature has shown that diagnosis is usually made very late after embolization has occurred, which will manifest with all the signs and symptoms associated with the loss of arterial integrity of the affected limb, that is, gangrene [4].

Multiple treatment options with varying degrees of invasiveness are available for the management of distal abdominal aortic thrombus, although established guidelines are still lacking [4]. Currently, there are three management approaches: conservative, endovascular intervention, and surgery. A review done by Quynh et al. (2022) showed that conservative management involving thrombolysis such as (streptokinase and alteplase) was most frequently used for the management of the condition [4]. Further review showed that the choice of endovascular management such as the use of stenting and intravascular ballooning depends on the clinical condition of the patient, failure of initial conservative therapy, and recurrent thrombus after surgical intervention [7, 8, 9, 10]. Surgical thrombectomy was recommended if the thrombus was found to be occluding large vessels and there was a deterioration in the patient's clinical condition despite initial conservative pharmacological management. The location and mobility of the thrombus were also factors that affected the choice of treatment between surgery and an endovascular approach. More distal thrombi, such as those affecting the distal abdominal aorta, were found to be better for surgical rather than endovascular intervention [11, 12].

## Author Contributions

**Hilary Chipongo:** conceptualization. **Abizer Sarkar:** visualization, writing – original draft. **Esmail Sangey:** supervision. **Kishan Chudasama:** writing – review and editing. **Amar Swali:** writing – original draft. **Shaffin Rajan:** writing – original draft, writing – review and editing.

## Ethics Statement

The case report was conducted following ethical standards, and patient confidentiality was maintained.

## Consent

Written consent for publication was obtained, and if needed, may be provided to the editor‐in‐chief.

## Conflicts of Interest

The authors declare no conflicts of interest.

## Data Availability

The data used to support the findings of this study are available from the corresponding author upon reasonable request.
